# Application of endobronchial ultrasound-guided transbronchial needle aspiration in the diagnosis and treatment of mediastinal lymph node tuberculous abscess: a case report and literature review

**DOI:** 10.1186/s13019-020-01360-3

**Published:** 2020-11-23

**Authors:** Yong Fang, Liping Cheng, Junhong Guo, Chunyan Wu, Ye Gu, Xiaofang You, Wei Sha

**Affiliations:** 1 Clinic and Research Center for Tuberculosis, Shanghai Key Laboratory of Tuberculosis, Shanghai Pulmonary Hospital, Tongji University School of Medicine, Shanghai, 200433 P.R. China; 2 Department of Pathology, Shanghai Pulmonary Hospital, Tongji University School of Medicine, Shanghai, P.R. China; 3 Department of Endoscope, Shanghai Pulmonary Hospital, Tongji University Schoo1 of Medicine, Shanghai, 200433 P.R. China; 4 Department of Imaging, Shanghai Pulmonary Hospital, Tongji University School of Medicine, Shanghai, 200433 P.R. China

**Keywords:** Mediastinal lymph node tuberculous abscess, Endobronchial ultrasonography, Transbronchial needle aspiration, Diagnosis, Treatment

## Abstract

**Background:**

This study aimed to report the experience of diagnosis and treatment of one rare case of mediastinal lymph node tuberculous abscess (MLNTA) using endobronchial ultrasound-guided transbronchial needle aspiration (EBUS-TBNA).

**Case presentation:**

An 18-year-old female patient was hospitalized in the Affiliated Hospital of Xuzhou Medical University in November 2017, due to intermittent left chest pain. She was suspected of infecting tuberculosis (TB) and thus received anti-TB treatment. Since April 1, 2018, she began to exhibit symptoms of chest distress. The patient was then admitted to Shanghai Pulmonary Hospital and continued receiving systemic anti-TB treatment during the whole course. On April 11, 2018, she received EBUS-TBNA to puncture pus and inject isoniazid. Simultaneously, the pus was sent for cytopathological and bacteriological examination, both supporting the diagnosis of TB in the patient. On April 24 and May 10, she received two times of EBUS-TBNA treatment. The symptoms of chest distress were relieved, but granulomatous neoplasm occurred at the EBUS-TBNA site on the trachea wall. The patient then received local clamp removal and cryotherapy on May 29 and Jul 19, respectively. Chest computed tomography (CT) reexamination on September 28 revealed that the MLNTA lesion had been completely absorbed, and electronic bronchoscopic reexamination on September 30 demonstrated that the granulomatous neoplasm on the trachea wall was entirely invisible.

**Conclusions:**

Using EBUS-TBNA to puncture and aspirate pus and inject drugs can be effectively used to diagnose and treat MLNTA, which provides a new, less invasive, safe and reliable method for diagnosis and treatment of MLNTA.

## Background

Tuberculosis (TB), a common chronic infectious disease caused by the bacillus *Mycobacterium tuberculosis*, is a worldwide major problem of public health [1]. Particularly, China makes up over 10% of the global TB burden [2]. TB typically affects lungs and it also spreads to extrapulmonary sites (extrapulmonary TB). Lymph node TB (LNTB) is most frequently in extrapulmonary TB, and particularly, TB can affect mediastinal lymph nodes, referring to mediastinal LNTB [3]. The mediastinal lymph node tuberculous abscesses (MLNTAs) are secondary to MLNTB, which are relatively rare. Caseous degeneration and necrosis, and liquefaction of some MLN may cause local abscesses, and enlarged lymph nodes and abscesses can compress the adjacent organs, e.g. trachea, bronchus, to induce external compressive stenosis, which further leads to symptoms of chest tightness and shortness of breath. Too much compression by enlarged lymph nodes and abscesses may break through bronchial wall to make rupture reach bronchus to form broncholymphatic fistula or break through the mediastinal pleura to let rupture spread in the lung to form abscesses, thus accelerating the spread and aggravating the disease.

Abscesses are short of blood supply, which hampers the delivery of the administered drugs. As a result, systematic chemotherapy may not achieve satisfactory outcomes. Therefore, suitable adjunctive intervention therapy is essential to relieve the related symptoms and control the spread of abscess. Endobronchial ultrasound-guided transbronchial needle aspiration (EBUS-TBNA) can determine the sites of lesions and vessels under the guiding of EBUS, accurately puncture to aspirate tissue specimens, pus, etc. and inject drugs in lesions [4]. EBUS-TBNA has recently been developed as a minimally invasive and safe procedure for the diagnosis of mediastinal lymphadenopathy such as MLNTB [5–8], mediastinal lymphoma [9], and mediastinal staging of lung cancer [10], with satisfactory safety and accuracy. However, the application of EBUS-TBNA in the diagnosis and treatment of MLNTA has not been reported yet. In this study, we showed our recent experience of using EBUS-TBNA to successfully diagnose and treat MLNTA in one patient, which achieved satisfactory outcomes.

## Case presentation

An 18-year-old female patient (school student) was hospitalized in the Affiliated Hospital of Xuzhou Medical University (Xuzhou, Jiangsu, China) in November 2017, due to intermittent left chest pain. Chest computed tomography (CT) showed left pleural effusion. Hydrothorax analysis demonstrated adenosine deaminase activity of 67 U/L and T cell test positive for TB infection, which suggested high possibility of TB infection. She thus received anti-TB treatment, including 0.3 g isoniazid (INH) (quaque die, qd), 0.6 g rifampicin (qd), 0.5 g pyrazinamide (ter in die, tid), and 0.75 g ethambutol (qd).

**Fig. 1:**
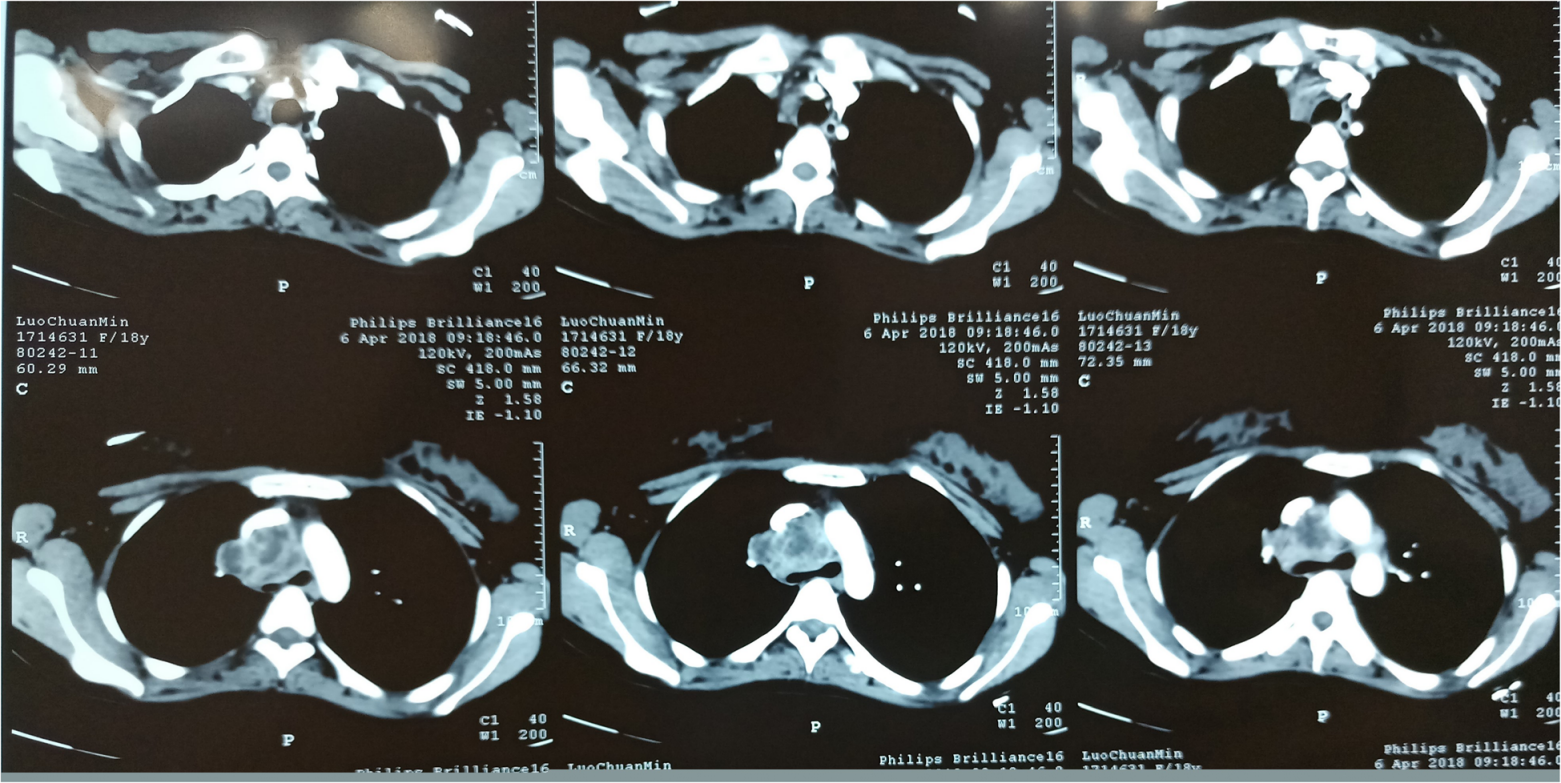
Chest CT reexamination on April 6, 2018

On January 14, the patient was hospitalized again in the initial hospital for repeated fever. During the treatment, she was found enlargement of supraclavicular and mediastinal lymph nodes, hypoechoic nodules outside the envelope of the left external lobe of liver (suggestive of involvement of lymph nodes), splenomegaly, multiple hypoechoic area within spleen (suggestive of TB), and a small amount of pelvic effusion. On February 2, she received supraclavicular lymph node puncture for histological observation, which showed many neutrophils, lymphocytes and phagocytes and a few epithelioid cells. This indicated that the possibility of scrofula could not be excluded. Since April 1 she began to exhibit symptoms of chest distress. The chest CT reexamination on April 6 revealed patchy and nodular shadow with increased density in the right upper lung, enlarged lymph nodes in the mediastinum and right supraclavicular region, with uneven enhancement, and necrotic areas (liquefaction area) in the interior. Enlarged mediastinal lymph nodes obviously compressed to narrow the trachea. The main bronchus on both sides were clear, and no obvious effusion was observed in the thorax on both sides (Fig. 1).

The patient was then admitted to Shanghai Pulmonary Hospital (Shanghai, China) for further treatment. After admission, the patient underwent physical examination: temperature 37 °C, pulse 80 times/min, respiration 18 times/min, and blood pressure 130/80 mmHg. She had a clear consciousness, without cyanosis in the mouth and lips, and had thick breath sound from both lungs, without obvious dry and wet rales. The heart rate was 80 times/min and heart rhythms were regular. Abdomen was smooth and soft, liver and spleen did not reach below costal, and pathological sign was (−). No edema was seen in both lower limbs. Blood gas analysis for pulmonary function on April 9, 2018 showed: pH 7.41, partial pressure of carbon dioxide 39.4 mmHg, oxygen partial pressure 108 mmHg↑, total hemoglobin 11.1 g/dL↓, oxygen saturation 99.2%, standard base excess 0.6 mmol/L, actual base excess 0.6 mmol/L, actual bicarbonate 24.7 mmol/L, standard bicarbonate 25.0 mmol/L, and alveolar artery oxygen partial pressure difference 0.0 mmHg ↓. Blood routine analysis on April 10, 2018 were as follows: hemoglobin 106.0 g/L↓, red blood cells 4.11*10^12^/L, white blood cells 5.19*10^9^/L, neutrophils % 66.3, lymphocytes % 21.4, monocytes % 9.6↑, eosinophils % 2.5, basophils % 0.2, absolute neutrophils amount 3.44*10^9^/L, absolute lymphocyte amount 1.11*10^9^/L, absolute monocytes count 0.50*10^9^/L, platelets 269*10^9^/L, hematocrit 0.323 L/L↓, mean red blood cell volume 79 fL↓, and mean hemoglobin content 26 pg↓. Blood biochemical assay on April 10, 2018 showed: r-glutamine transferase 42 U/L, alanine aminotransferase 13 U/L, aspertate aminotransferase 27 U/L, aspertate aminotransferase isoenzyme 6 IU/L, alkaline phosphatase 87 U/L, total bilirubin 11.0 μmol/L, direct bilirubin 3.6 μmol/L, total bile acid 2.9 μmol/L, total protein 78 g/L, albumin 42 g/L, globulin 35 g/L, albumin/globulin 1.2 ↓, prealbumin 185 mg/L ↓, α- L-fucosidase 17 U/L, uric acid 530 μmol/L ↑, urea nitrogen 3.3 mmol/L, creatinine 43 μmol/L↓, glucose 5.2 mmol/L, potassium 3.6 mmol/L, sodium 139 mmol/L, chlorine 106 mmol/L, calcium 2.40 mmol/L, and phosphorus 1.52 mmol/L.

**Fig. 2:**
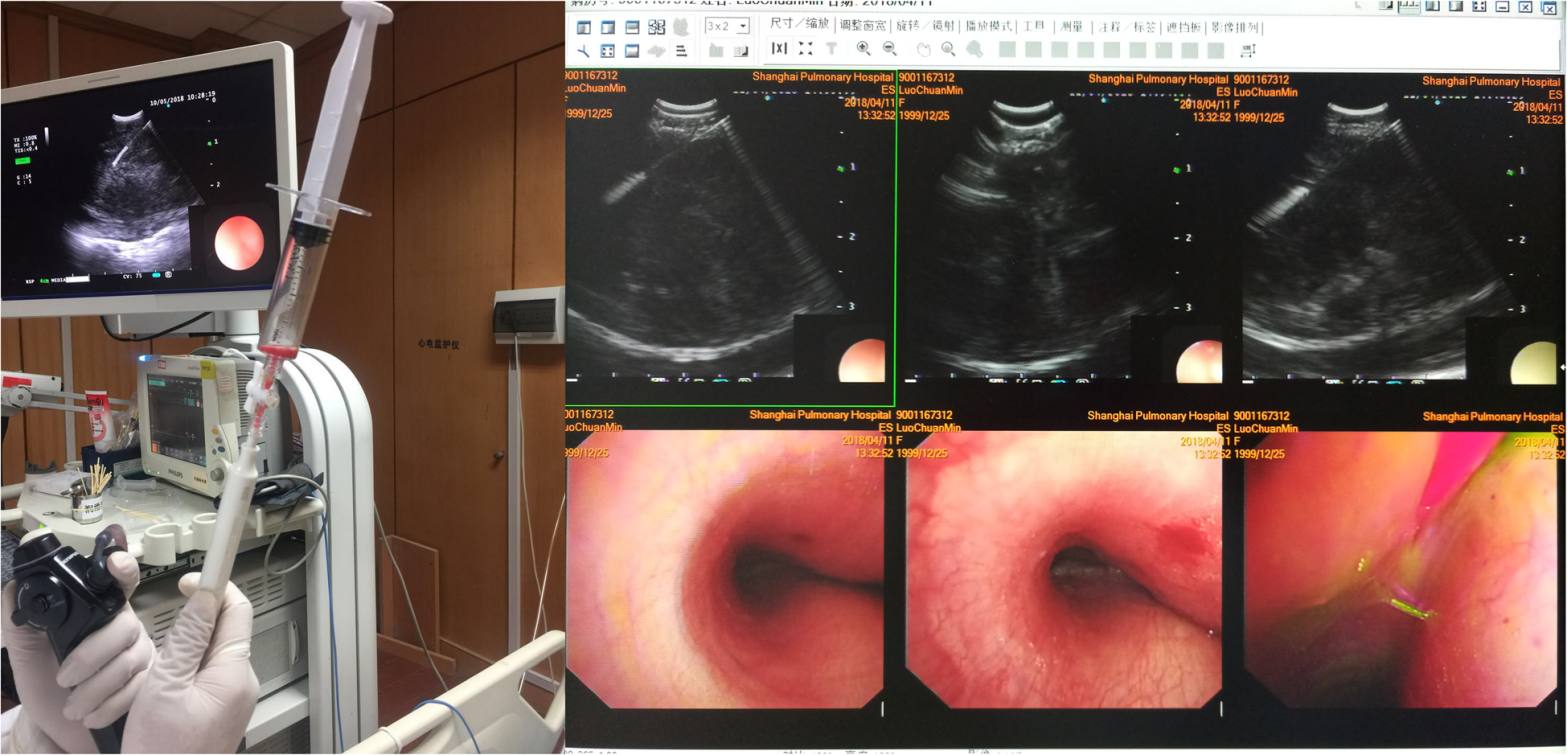
Pus was punctured and aspirated through EBUS-TBNA on April 11, 2018

The patient continued receiving systemic anti-TB treatment during the whole course, including 0.3 g INH (peros, po/qd), 0.6 g rifampicin (po/qd), 0.75 g ethambutol (po/qd), and 0.5 g pyrazinamide (po/tid). All the drugs were purchased from the Shanghai Sine Pharma (Shanghai, China). On April 11, she received EBUS- TBNA treatment, namely, EBUS examination, with the help of an ultrasonic tracheoscope (UMBS20-26R, Olympus, Shinjuku, Tokyo, Japan) and the corresponding EU-C2000 ultrasound processing apparatus (Olympus), to observe the enlarged mediastinal lymph nodes at the Four Right group, followed by puncturing and aspirating all the pus (about 5 ml) from the abscess with a specific 21-G needle (NA-201SX-4022, Olympus) (Fig. 2). Then 0.1 g INH injection (Southwest Pharma, Chongqing, China) was administered through the needle during EBUS-TBNA. The pus underwent smear detection to show positive for acid bacillus and simultaneously, the pus was sent out for cytopathological examination. There was no obvious main complaint of discomfort from the patient after operation.

**Fig. 3:**
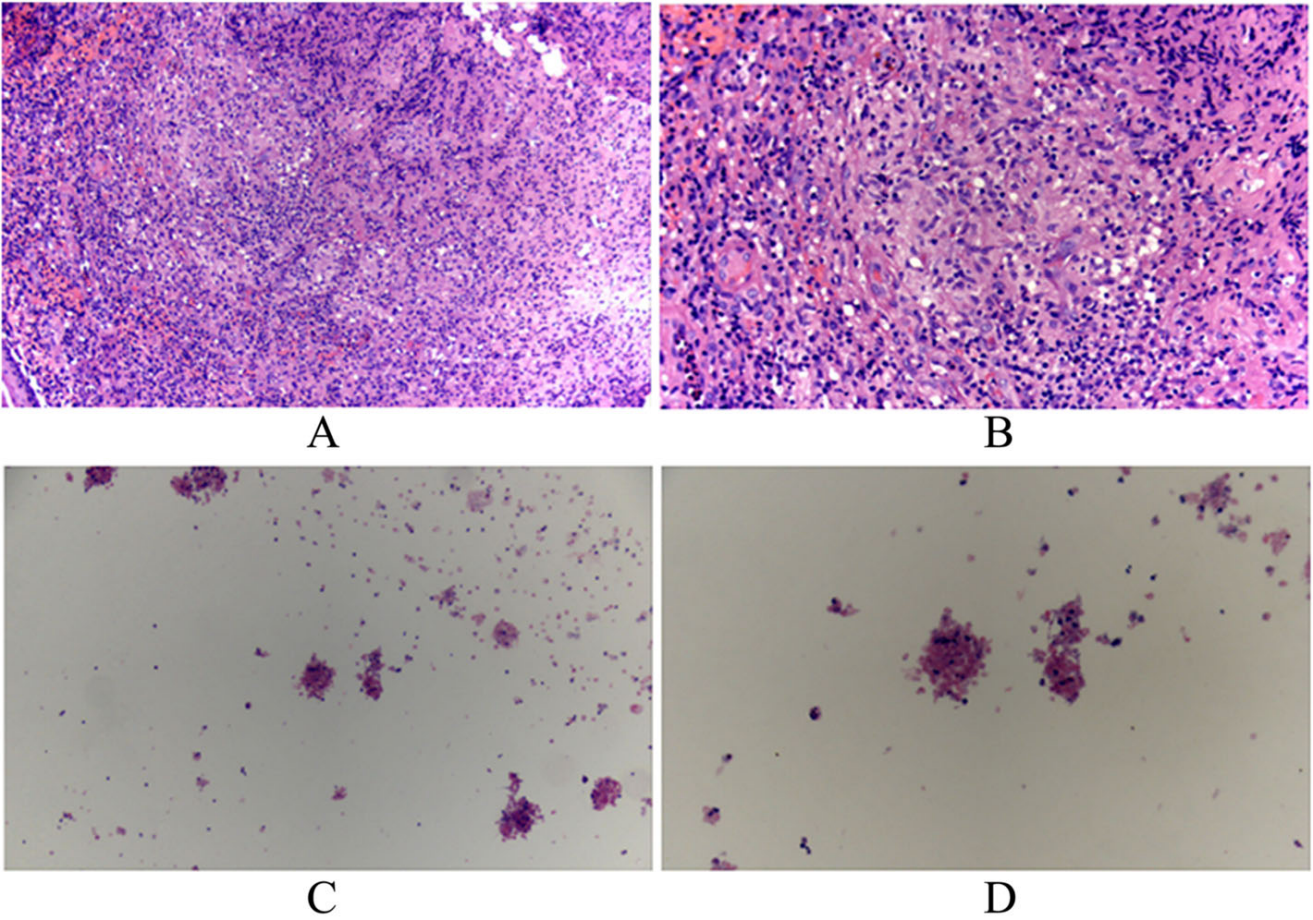
Cytopathological observation of the aspirated pus. **a**, **b** Hyperplasia of epithelioid cells and lymphocytes infiltration and granuloma was formed in the aspirated pus (A, X 100; B, X 200). **c**, **d** Necrotic cells and a few lymphocytes were observed in the aspirated pus (C, X 40, D, X100)

Cytopathological examination for the aspirated pus indicated inflammatory necrosis tissues and a few epithelioid cells (Fig. 3). Polymerase chain reaction (PCR) assay showed the pus sample was positive for the detection of prokaryotic 16S RNA gene, *RV0577* coding an important antigen RV0577 in TB patients [11, 12], and *IS6110* fragment, an insert fragment within TB [13, 14], indicating the presence of featured DNA fragments of TB. The smear examination of pus showed positive for acid bacillus. These indicate the infection of Mycobacterium TB in the patient. Collectively, the MLNTB was determined.

**Fig. 4:**
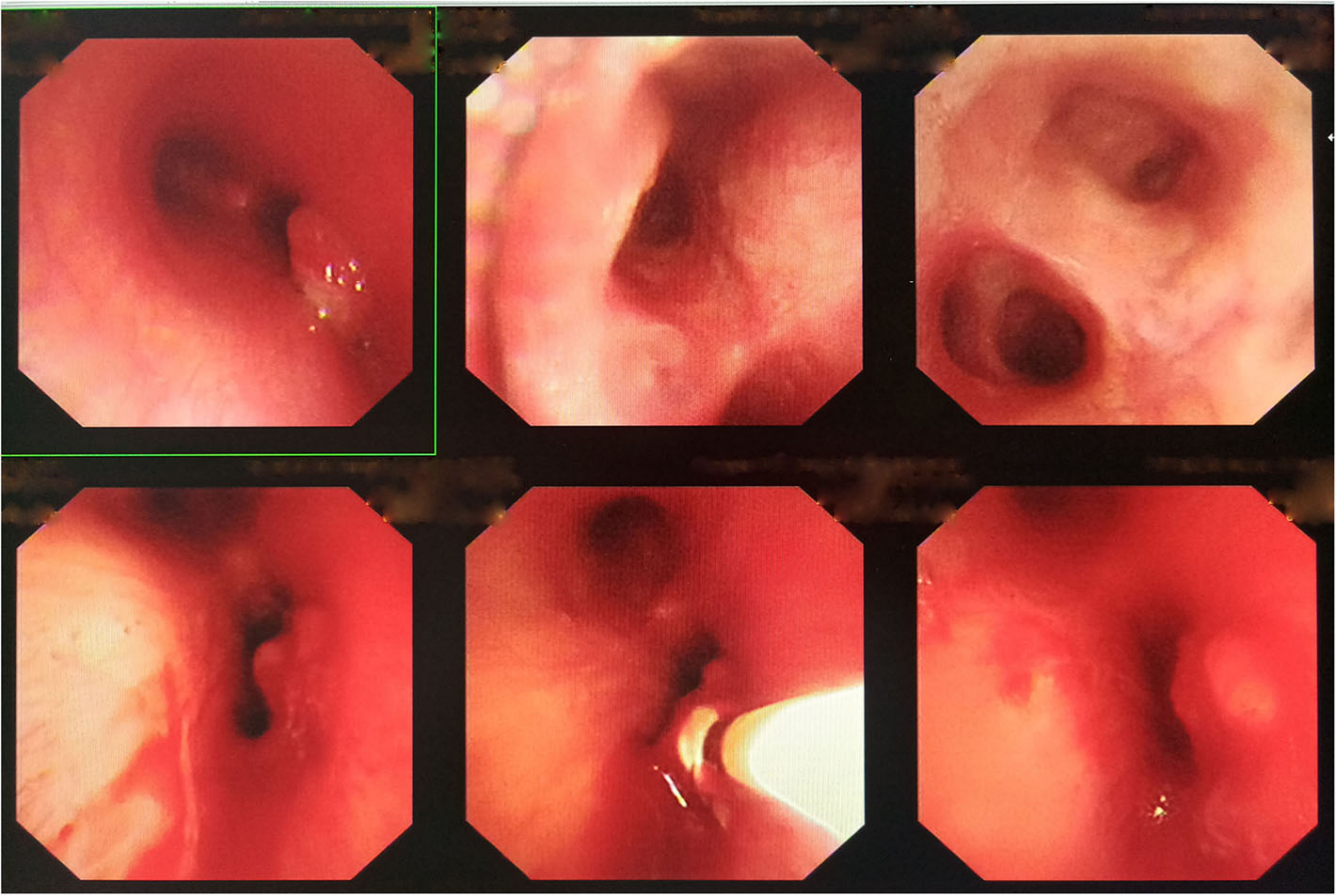
Electronic bronchoscopic examination on May 29, 2018

On April 24 and May 10, she received two times of EBUS-TBNA to puncture and aspirate pus, 2 ml for each time, and to administer 0.1 g INH injection each time. After that, the symptoms of chest distress were obviously relieved. On May 29, she received electronic bronchoscopic examination and was found granulomatous neoplasm at the EBUS-TBNA site on the trachea wall (Fig. 4).

**Fig. 5:**
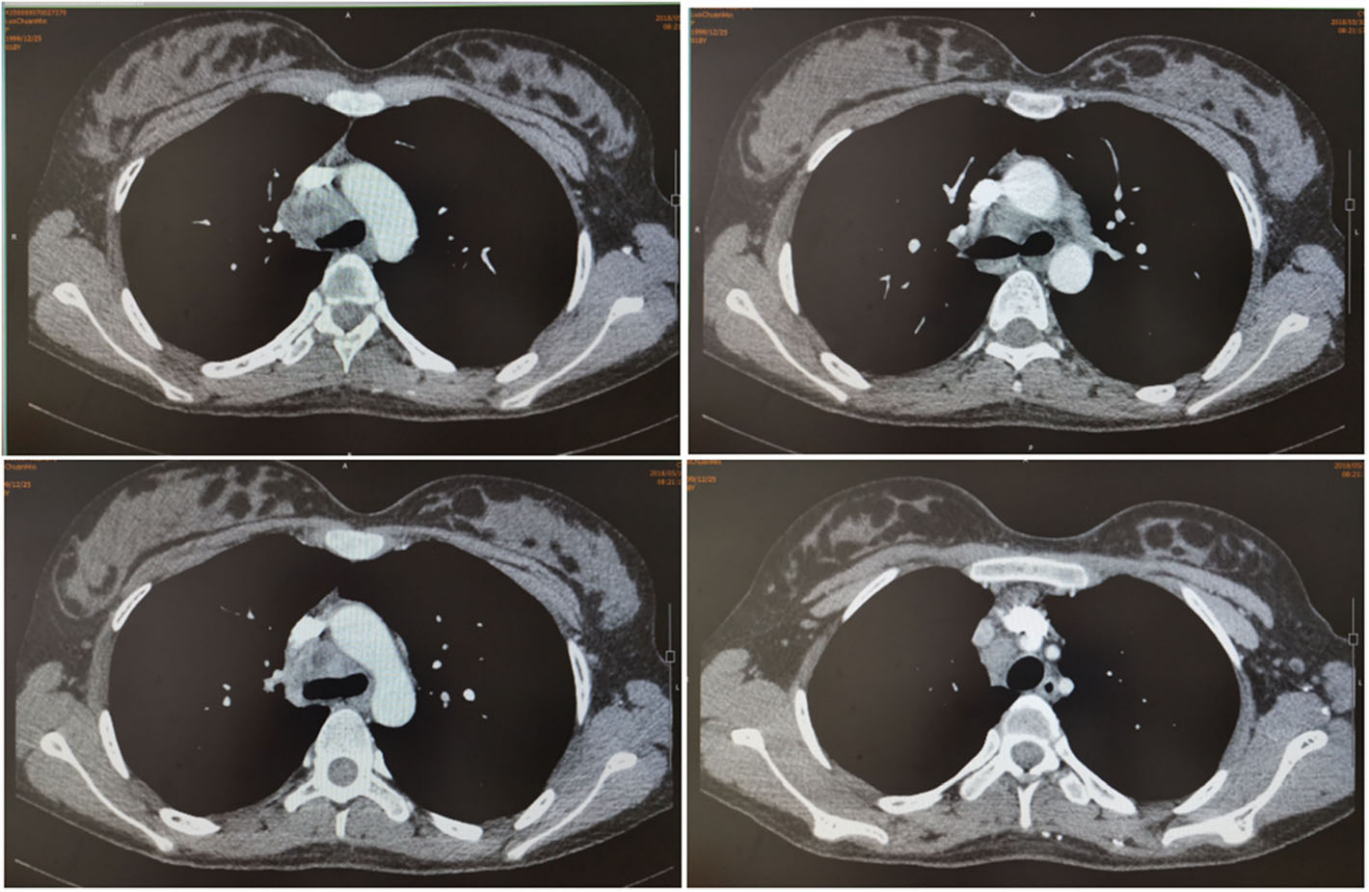
Chest CT reexamination on May 30, 2018

**Fig. 6:**
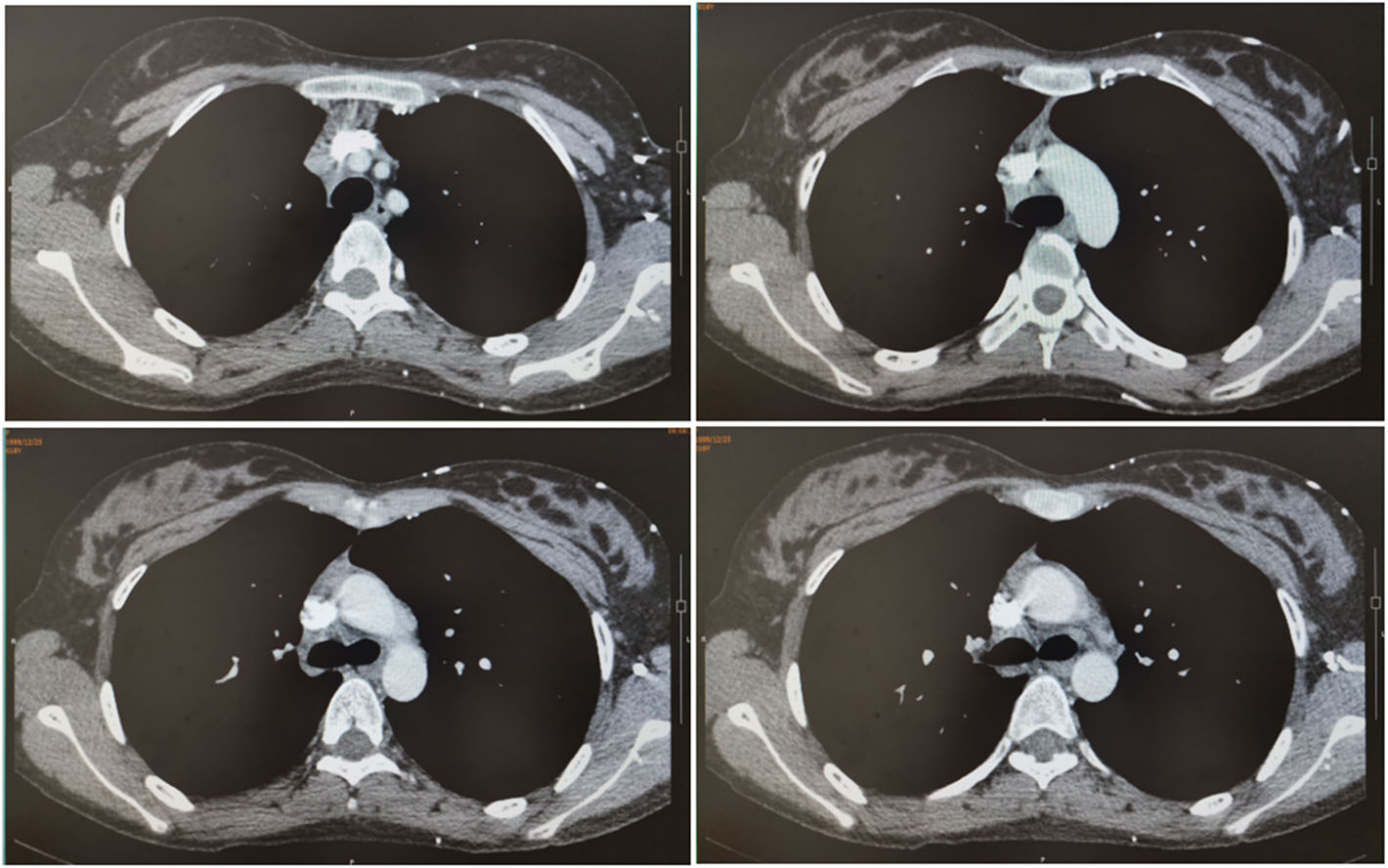
Chest CT reexamination on September 28, 2018

**Fig. 7:**
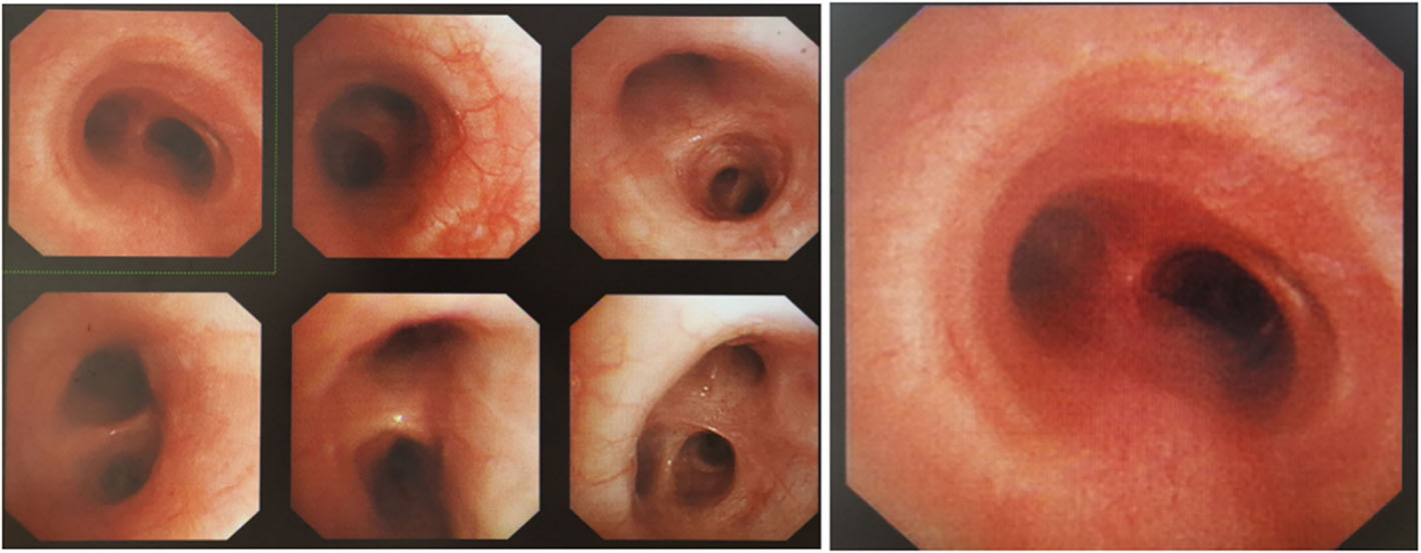
Electronic bronchoscopic reexamination on September 30, 2018

**Fig. 8:**
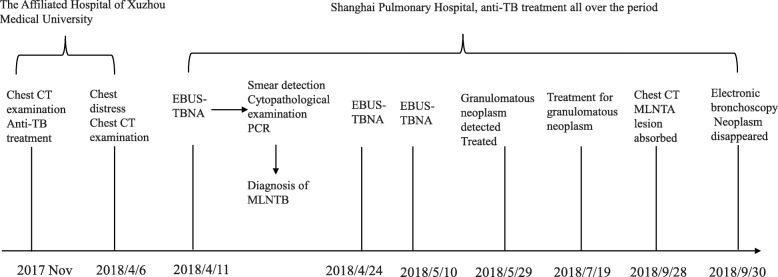
The timeline for the diagnosis and treatment of MLNTA in this patient

To treat the granulomatous neoplasm occurring after the EBUS-TBNA procedure, the patient then received local clamp removal treatment and cryotherapy for 90 s after EBUS-TBNA on May 29 and Jul 19, respectively, by using a K320 cryosurgery treatment machine (Beijing Kooland Medical Equipment Co., Beijing, China), with the probe temperature of − 40 ~ − 75 °C. Chest CT reexamination on May 30 indicated the MLNTA lesion was obviously decreased (Fig. 5). Chest CT reexamination on July 17 revealed that the MLNTA lesion had been basically absorbed, and that on September 28 confirmed that the MLNTA lesion had been completely absorbed (Fig. 6). Electronic bronchoscopic reexamination on July 19 showed the granulomatous neoplasm at the EBUS-TBNA site on the trachea wall was obviously decreased, and that on September 30 demonstrated that the granulomatous neoplasm on the trachea wall was entirely invisible (Fig. 7). At this point, the symptoms of chest distress were completely disappeared, and the patient achieved satisfactory treatment. The timeline for the diagnosis and treatment of MLNTA in this patient was summarized in Fig. 8.

This study was approved by the Ethics Committee of the Shanghai Pulmonary Hospital, Tongji University School of Medicine. Informed consent was obtained from the patient.

## Discussion

EBUS-TBNA is a minimally invasive and safe procedure for the assessment of mediastinal lymphadenopathy [5–10]. We searched the Web of Science and PubMed databases for references using “mediastinal lymph node”, “tuberculosis” and “endobronchial ultrasound-guided transbronchial needle aspiration”. As of June 20, 2020, using these above key words searched 67 results in PubMed, and the main results were summarized below.

Mediastinal TB is rare and a diagnostic challenge. EBUS-TBNA has been used to sample for analysis in the diagnosis of MLNTB in Chinese [7, 15], Indian [16, 17] and other populations. For example, Gulla et al. used EBUS-TBNA in the evaluation of mediastinal pathologies, including TB [17]. Dhamija et al. showed high sensitivity (97.6%) and specificity of applying EBUS-TBNA in the diagnosis of mediastinal TB [18]. With the help of EBUS-TBNA, Ge et al. identified a rare case of systemic lymph nodes TB which was previously misdiagnosed as lymphoma [19], Ayub et al. differentiated benign from malignant nodes in patients with intrathoracic lymphadenopathy, including diagnosis of TB [20], Lyn et al. even determined multidrug-resistant TB of a mediastinal lymph node in a patient [21]. Lin et al. employed EBUS-TBNA rinse fluid to diagnose mediastinal TB lymphadenitis by means of polymerase chain reaction for *Mycobacterium tuberculosis* (TB-PCR) [22]. Until now, it seems that the application of EBUS-TBNA was just focused on the diagnosis instead of treatment of MLNTB.

MLNTAs, secondary to MLNTB, are not common. There are very few studies reporting the treatment for MLNTAs. For example, Zuo et al. used video-assisted thoracoscopic surgery (i.e. radical debridement and drainage of abscesses, followed by removal of intrathoracic lesions) in combination with anti-TB chemotherapy for MLNTAs, leading to good recovery in patients but causing some complications (such as recurrent laryngeal nerve injury and poor wound healing) in some cases [23]. In this study, we admitted a young female patient and used EBUS-TBNA to puncture and aspirate pus sample for cytopathological examination and PCR detection, which confirmed the diagnosis of MLNTB in the patient. As shortage of blood supply by vessels in abscesses, chemotherapeutic drugs cannot easily reach abscesses, which may be the reason why the patient could not turn better after the systematic chemotherapy in the previous hospital. In our hospital, in addition to systematic anti-TB treatment, we used EBUS-TBNA to determine the sites of abscesses to accurately and effectively administer INH. The treatment scheme eventually achieved satisfactory, i.e. the MLNTA lesion had been totally absorbed. Of note, systematic anti-TB chemotherapy was simultaneously carried out, which can effectively decrease the risk of iatrogenic secondary infection to control any infection-related complications.

Although EBUS-TBNA has been successfully used for the diagnosis of mediastinal lymphadenopathy such as MLNTB [5–10], there have no studies reporting the application of EBUS-TBNA in the diagnosis and/or treatment of MLNTAs yet. This may be the first report introducing the employment of EBUS-TBNA to puncture and aspirate pus for diagnosis of MLNTAs and administer drugs to treat MLNTAs. Worth of mention, about 20 days after the last EBUS-TBNA treatment (May 10, 2018), the patient was found granulomatous neoplasm at the EBUS- TBNA site on the trachea wall after electronic bronchoscopic examination (May 29). It was speculated that granulomatous neoplasm was a complication of the EBUS-TBNA treatment. However, whether or not granulomatous neoplasm is really a complication and what is the risk rate need to be further observed and validated after more patients are treated by EBUS-TBNA in the future. After two times of local clamp removal treatment and cryotherapy (on May 29 and Jul 19, respectively), the granulomatous neoplasm was disappeared evidenced by the electronic bronchoscopic reexamination (September 30), indicating the potential complication of EBUS-TBNA can be effectively controlled. Moreover, this is the first report about the application of EBUS-TBNA in the diagnosis and treatment of MLNTAs. With the accumulation of experience of more and more such surgeries later, the risk of granulomatous neoplasm can be further controlled.

If granulomatous neoplasm is really a complication of EBUS-TBNA, we suppose that the treatment times, means of puncturing and aspirating of pus from abscess, the needle (a specific 21-G needle was used in this study) whereby puncturing and aspirating are performed, etc. may be related to the risk of granulomatous neoplasm, which needs to be optimized to decrease the risk of this complication.

From our experience of one case of EBUS-TBNA for MLNTA, the indication could be initially supposed as follows: 1) patient exhibits symptoms of TB, 2) MLNTAs are detected in patient after imaging examination, which compress to narrow the trachea, 3) no significant improvement is observed in abscess after regular anti-TB treatment, and 4) lymph node lesions can be reached by EBUS. Of course, with more MLNTA patients receiving EBUS-TBNA in the future, the indication of the procedure will be further improved.

Compared with VATS for MLNTAs [23], EBUS-TBNA in this study may have some advantages, although both EBUS-TBNA and VATS may have complications which can be effectively controlled. The means of EBUS-TBNA is different from VATS in that the former invasively and precisely administers drugs in the MLNTA lesions to make them completely absorbed, while the latter tries to completely resect the abscess foci and infected/suspicious lymph nodes via VATS. Both the two surgical methods are promising, but theoretically for avoiding of resection, EBUS-TBNA might be less invasive and more suitable for patients with surgical contraindications in comparison with VATS. EBUS-TBNA even can be combined with other methods to further improve the treatment outcomes. In addition, in contrast to VATS that is mainly used for accurate resection, EBUS-TBNA can be used for not only treatment (administration of drugs in the lesions) but also diagnosis. Moreover, EBUS-TBNA costs much less than VATS. In this study we tried to employ EBUS-TBNA, a relatively common facility, to develop an invasive and effective means for MLNTAs, which appears to be more practical and popular in clinics when compared with VATS.

A limitation of this study is that since the cases of using EBUS-TBNA in the diagnosis and treatment of MLNTAs are very scarce, only one case is reported in this study. Prospective studies with more cases are warranted to validate the diagnostic and treatment values of EBUS-TBNA for MLNTAs.

## Conclusions

This study suggests a new, effective, less invasive and safe method for diagnosis and treatment of MLNTAs by using EBUS-TBNA to puncture and aspirate pus for diagnosis and inject drugs for treatment.

## Data Availability

All data generated or analyzed during this study are included in this published article. Besides, any additional data/files may be obtained from the corresponding author.

## Abbreviations

MLNTA: Mediastinal lymph node tuberculous abscess
EBUS-TBNA: Endobronchial ultrasound-guided transbronchial needle aspiration
TB: Tuberculosis
CT: Computed tomography
LNTB: Lymph node TB
MLNTA: Mediastinal lymph node tuberculous abscesses
INH: Isoniazid
qd: quaque die
tid: ter in die
po: peros
PCR: Polymerase chain reaction
